# Automation of a Beckman liquid scintillation counter for data capture and data-base management

**DOI:** 10.1155/S1463924688000203

**Published:** 1988

**Authors:** William Neil, Thomas J. Irwin, Joseph J. Yang

**Affiliations:** 1 Mobil Environmental and Health Science Laboratory P. 0. Box 1029 Princeton New Jersey 08540 USA; 2 Squibb E. R. & Sons, Inc. Medical Affairs P.O. Box 4000 Princeton New Jersey 08540 USA


Journal of Automatic Chemistry, Vol. 10, No. 2 (April–June 1988), pp. 106–109

### Automation of a Beckman liquid scintillation counter for data capture and data-base management

**William Neil†, Thomas J. Irwin* and Joseph J. Yang**
*Mobil Environmental and Health Science Laboratory, P.O. Box 1029, Princeton, New Jersey 08540, USA*

*A software package for the automation of a Beckman LS9000 liquid scintillation counter is presented. The package provides effective on-line data capture (with a Perkin Elmer 3230 32-bit minicomputer), data-base management, audit trail and archiving facilities. Key features of the package are rapid and flexible data entry, background subtraction, half-life correction, ability to queue several sample sets pending scintillation counting, and formatted report generation. A brief discussion is given on the development of customized data processing programs.*

$^{14}C$ and $^{3}H$ radioisotopes have been frequently used in metabolism studies of drugs and other xenobiotics. They are also useful in comparing the relative absorption of a given compound from different routes of administration. Incorporation of either or both radioisotopes in the test compound allows for highly specific and sensitive determinations of the test compound and metabolites in the body tissues and excreta.

Liquid scintillation counting (LSC) is the method of choice in measuring such weak beta-emitting radioisotopes as $^{3}H$ and $^{14}C$. The scintillation process involves interactions between a test sample containing beta emitters and the scintillation cocktail (a mixture of organic solvent and scintillator) leading to the emission of photons, which are measured by photomultiplier tubes in the LS counter.

Following a typical metabolism or bioavailability study in our laboratory, measurements of radioactivity in collected samples are effectively carried out with a Beckman LS9000 counter. It is equipped with three counting channels and with microprocessors that automatically correct for background and counting efficiency. However, the steps that follow which include data entry, record keeping, documentation, and quality assurance of numerous quantities of LSC data are laborious and time-consuming and in need of obvious automation.

Although LSC data capture software has been recently developed for use with laboratory personal computers [1], the availability of data management and application software has been limited. A review of the commercially available software packages did not provide reasonable solutions to our needs in data capture, data-base management and data processing. Thus, we have chosen to develop our own LSC automation system (LSCAS) which includes the following principal features:

(1) Automatic data capture—LSC data generated by the counter is captured on-line with a minicomputer.

(2) Flexibility—unlimited selections are available for sample identification.

(3) Reduction of record-keeping—data are clearly documented with proper sample identifications.

(4) GLP requirements—data cannot be edited without specific documentation.

(5) Efficient data management—data can be easily archived and retrieved.

(6) Elimination of data entry—data generated from the counter are automatically entered into the customized application programs for data processing.

This paper delineates our efforts in the automation of data capture and data-base management. Details on the development of application programs to perform customized processing of LSC data will be published in a separate paper.

### Materials and methods

*Computer equipment*

LSCAS consists of an Ergo CRT display terminal, a Perkin-Elmer 3230 32-bit minicomputer with a 300 megabyte disk, a Beckman LS9000 liquid scintillation counter equipped with a standard RS232 interface and a Perkin-Elmer CP100 printer. The Perkin-Elmer is linked to a VAX 11/750 using a VAX 3780 Protocol Emulator (Digital Equipment Corporation).

*Computer software*

The software package for LSCAS was written in FORTRAN 77 under Perkin-Elmer OS32 operating system. There are five major components in the package: data collection, queue maintenance, status reports, archive maintenance and application programs.

† To whom correspondence and requests for reprints should be addressed.
* Present address: Squibb E. R. & Sons, Inc., Medical Affairs, P.O. Box 4000, Princeton, New Jersey 08540, USA.

| | AVAILABLE PROGRAMS |
|---|---|
| 1 - INSTRUMENT #: 01 | 1) BIOAVAILABILITY |
| 2 - PROGRAM #: 04 USER #: 04 | 2) IN VITRO PERCUTANEOUS ABSORPTION |
| 3 - STARTING POSITION #: 0020 | 3) HISTOGRAM GENERATION |
| 4 - ENDING POSITION #: 0034 | 4) OXIDIZER EFFICIENCY |
| 5 - DATE-OF-INITIATION: 11/11/11 | 5) LSC CALIBRATIONS |
| 6 - APPLICATION PROGRAM #: 01 | 6) WIPE TESTS |
| 7 - INITIALS: WN | 7) BIODEGRADATION |
| 8 - DATE-OF-RUN: | |
| 9 - DATE ARCHIVED: | |

*Figure 1. Sample scheduling data entry.*

| SEQ# | POS# | ID# | SAMPLE DESCRIPTION | SAMPLE COMMENTS | BKGRD | #R |
|---|---|---|---|---|---|---|
| 001 | 020 | 010-111 | BACKGROUND SAMPLE | | | 01 |
| 002 | 021 | 010-111 | BACKGROUND SAMPLE | | | 02 |
| 003 | 022 | 010-111 | BACKGROUND SAMPLE | | | 03 |
| 004 | 023 | 010-112 | FECES 24 HR | | 001-003 | 01 |
| 005 | 024 | 010-112 | FECES 24 HR | | 001-003 | 02 |
| 006 | 025 | 010-112 | FECES 24 HR | | 001-003 | 03 |
| 007 | 026 | 010-113 | FECES 48 HR | | 001-003 | 01 |
| 008 | 027 | 010-113 | FECES 48 HR | | 001-003 | 02 |
| 009 | 028 | 010-113 | FECES 48 HR | | 001-003 | 03 |
| 010 | 029 | 010-112 | URINE 24 HR | | 001-003 | 01 |
| 011 | 030 | 010-112 | URINE 24 HR | | | 02 |
| 012 | 031 | 010-113 | URINE 24 HR | SAMPLE SLIGHTLY RED | | 01 |
| 013 | 032 | 010-113 | URINE 24 HR | SAMPLE SLIGHTLY RED | | 02 |
| 014 | 033 | 010-111 | URINE 48 HR | | | 01 |
| 015 | 034 | 010-111 | URINE 48 HR | | | 02 |

*Figure 2. Sample identification data entry. After the data in figure 1 has been entered, the user is provided with properly sequenced position numbers. Positive sample identification then occurs by entering the data that corresponds to its position in the liquid scintillation counter.*

### Data collection

### Data entry

Procedures for entering sample identifications into LSCAS are divided into two parts as shown in figures 1 and 2. Three useful features were included into this portion of the program: (1) sequencing of sample position numbers occurs automatically as a result of the STARTING POSITION and ENDING POSITION parameters entered (figure 1). This saves the user from having to enter the position number for each individual sample; (2) SAMPLE COMMENTS (figure 2) can be used to describe sample characteristics and the generated records serve as an electronic notebook; and (3) automatic data entry is performed for replicates by entering the numerical value that corresponds to the number of replicates (# R in figure 2). Using triplicate as an example, information on the sample identification needs to be entered only for the first sample; identical information is automatically entered for two other samples.

A rapid and flexible data entry system is especially useful in LS counter automation. It is often desirable to add, delete or change descriptions from the menu. A predefined menu is extremely restrictive in a laboratory where changes are constantly being made on existing test procedures. An alternative of a free text entry for each sample provides flexibility but significantly increases the time required for data entry. Therefore, a second alternative was chosen which provides more efficiency. An unlimited number of user defined menus (figure 3) created using the text editor are implemented by entering a row of sample descriptions in the form of a template file. The program searches for the menu in the user's account providing a default menu in its absence.

The option to perform free text entries is still available by providing an additional selection (figure 3) DEFINE SITE, which will prompt the user for the sample description.

SELECTION MENU

| | | |
|---|---|---|
| 01) URINE 24 HR | 15) FECES 120 HR | 29) MUSCLE |
| 02) URINE 48 HR | 16) CAGE WASH | 30) FAT |
| 03) URINE 72 HR | 17) HEART | 31) BONE |
| 04) URINE 96 HR | 18) BRAIN | 32) SKIN |
| 05) URINE 120 HR | 19) SMALL INTESTINE | 33) RESIDUAL CARCASS |
| 06) URINE WASH 24 HR | 20) LARGE INTESTINE | 34) SKIN WIPE |
| 07) URINE WASH 28 HR | 21) LIVER | 35) CHARCOAL TUBE |
| 08) URINE WASH 72 HR | 22) KIDNEY | 36) CELL |
| 09) URINE WASH 96 HR | 23) SPLEEN | 37) TAPE |
| 10) URINE WASH 120 HR | 24) STOMACH | 38) BLOOD |
| 11) FECES 24 HR | 25) TESTES | 39) FETUS |
| 12) FECES 48 HR | 26) OVARIES | 40) PLACENTA |
| 13) FECES 72 HR | 27) BLADDER | 41) DOSE |
| 14) FECES 96 HR | 28) LUNG | 42) CO2 TRAP SOLUTION |
| | | 43) PIPET RINSE |
| | | 44) SPATULA RINSE |
| | | 45) DEFINE SITE |

ENTER ITEM NUMBER : ___

*Figure 3. Sample selection menu. The number of sample description selections are unlimited due to the user-defined selection menus. Free text entry is also provided (option 45).*

### Data capture

The most vital section of LSCAS is its data capture program. Radioactivity values generated from the counter must be correctly matched with the corresponding sample identifications. Figure 4 graphically illustrates the algorithm that performs this function.

Basically, the LSC process is performed in the following fashion. Vials containing test samples and scintillation cocktail are placed in consecutive order into the counter in a sample conveyer train. Each sample has a specific position number corresponding to its location in the train. In the position immediately preceding the first vial of each sample set is a COMMAND TOWER. The

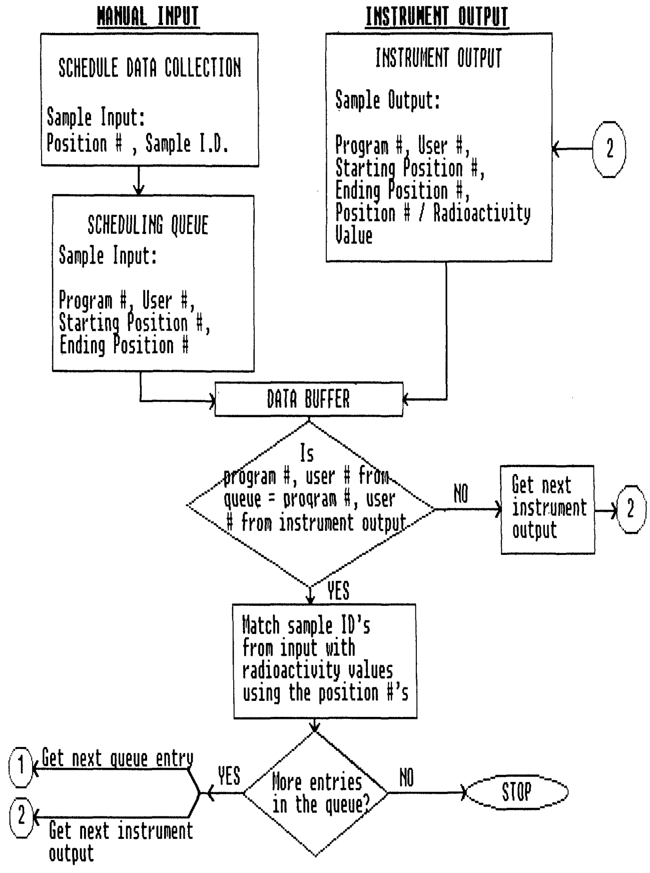

*Figure 4. Flowchart of data capture process.*

COMMAND TOWER contains four dials corresponding to the numbers 1, 2, 4, and 8. By dialling the appropriate combination of numbers, the user can select up to 10 counting programs (for example dialling 1, 3, and 4 selects program 8). The COMMAND TOWER is read by a photoelectric cell and instructs the counter what LSC program will be used for subsequent countings (for example isotope(s) counted, counting time). Only one COMMAND TOWER needs to be used if all samples are to be counted under the same program. A STOP TOWER (no dial settings) is placed directly after the last sample in the train to terminate the counting. All LSC data generated are sent via the RS232 interface to the Perkin-Elmer 3230 minicomputer where the matching of radioactivity values with sample identifications takes place.

*Background subtraction*

Radiation originating from the sample vial, the scintillation cocktail, or instrumental sources can result in low level background counts. These counts must be subtracted from the sample count, especially when analysing samples with minute amounts of radioactivity. Although the Beckman LS9000 counter allows a fixed background value to be subtracted from the LSC data of samples analysed, it is more desirable to perform background subtractions based on values obtained from sample blanks prepared specifically for the corresponding sets of samples. In LSCAS, this latter approach is accomplished by placing the sample blanks in consecutive order into the sample conveyer train and supplying the program with the position number of sample blanks (figure 2).

*Half-life corrections*

LSC of radioisotopes, especially those with short half-life (for example $^{32}P$, $^{125}I$), requires correction factors due to loss of inherent radioactivity through decay. Half-life corrections are easily performed on late model LS counters by supplying the counter with information on the time lag between a specified date and the analysis date. This feature is not available on the Beckman LS9000 counter, therefore a separate step has been included in LSCAS to perform similar automatic half-life corrections.

*Data documentation*

In order to meet requirements of Good Laboratory Practices (GLP), LSCAS was designed with an AUDIT TRAIL feature which documents any changes made to the raw data regarding who, what, where, when and how. In LSCAS, a user cannot modify any radioactivity data generated originally by the counter but can change entries such as sample identification number provided that appropriate reasons are given. The AUDIT TRAIL file can be examined at any time as shown in figure 5.

AUDIT TRAIL FOR FILE #F84JR5.DAT

| DATE | INITIALS | CODE | REASON |
|---|---|---|---|
| 01/25/86 | WN | 0 | ENTERED INCORRECT PROJECT # PROJECT #50101 WAS CHANGED TO #51011 |
| 01/25/86 | WN | 0 | ENTERED INCORRECT ID # POSITION #125 ID #000-101 WAS CHANGED TO ID #000-102 |

*Figure 5. Typical Audit trail file.*

The raw data in LSCAS are also protected with the following measures: (1) each sample data set is assigned a unique data file name and the data file is placed into a protected account, separate from the user's account, to prohibit editing by the user. (2) Users are not allowed to access the LSC data from another user's account. This is accomplished by searching through a master data file (containing the file history of all collected sample sets) for the account number under which the data was saved. This account number must match the account number of the user currently seeking to access the data.

*Queue maintenance*

QUEUE MAINTENANCE allows sample data sets to be placed on a 'waiting list' (queue) in LSCAS pending LSC counting. After scheduling the sample sets to be counted under data collection, they must then be placed into the queue. Each entry into the queue represents a sample data set under one COMMAND TOWER and as a result, the user does not need to individually start the automation process for each sample data set. This setup is extremely useful when counting sets of samples overnight allowing the (automation) process to operate continuously.

BECKMAN LS9000 LIQUID SCINTILLATION COUNTER SERIAL # 7772224

ANY TITLE CAN BE PLACED HERE

RADIOACTIVITY DATA

*Figure 6. Sample report showing instrument generated data merged with manually entered sample identifications.*

Samples can also be appended to the queue, moved from the present location in the queue to a new location, inserted between two sample sets, or deleted from the queue. Additionally, high priority samples can be placed before low priority ones by simply changing the order in which the sample data sets are to be counted. There is no need to physically change the location of samples in the counter.

### *Status reports*

Raw LSC data from the counter along with manually entered sample identifications can be displayed on the CRT or a hard copy device using the STATUS REPORTS option. The users are also provided with options to retrieve specific information quickly and easily on all data meeting a single criterion. For example, the user can retrieve all LSC data generated on 12 October, 1986 and 6 January to 3 March, 1986 by entering 10/12/86 and 01/06/86\03/03/86 in LSCAS, respectively.

The format of a typical report (figure 6) consists of three parts: header information, instrument parameters and raw data. Each page of the report is labelled with name and serial number of the counter used, date printed, project number, an optional title and page number. Page numbers are included to ensure that each page of the data from a sample data set is accounted for in the event that the data become separated. The header page includes information such as analysis date, account number, initials of the individual performing the work and a place for the signature of a separate person who reviews the data. The instrument parameter page contains all of the instrument parameters used in the LSC. The raw data pages list all LSC data generated along with corresponding sample identification descriptions.

### *Archive maintenance*

An efficient archiving method is available in LSCAS which allows data to be transferred periodically from disk to magnetic tape. This method is needed because of rapid build-up of LSC data on the disk from day-to-day operation. Users can selectively submit batches of completed sample data sets to the in-house computer system manager for archives. Files corresponding to the desired sample data set are placed in a queue along with their history. Attempts to access information from an archived data file will result in a message stating that information requested has been archived.

### *Application programs*

This menu selection serves primarily to format the data as ASCII text files before data transfer to the VAX 11/750. The three exceptions are the HISTOGRAM GENERATION, LSC CALIBRATIONS and WIPE TESTS programs which reside on the Perkin-Elmer (see figure 1).

Recently, the HISTOGRAM GENERATION program was completed which generates histograms (bar charts) of radioactivity value versus sample description. The data representing the $y$ variables is entered by using a predefined menu or choosing the DEFINE SITE menu option (see figure 5). The data must be entered with numeric data preceding any text. The program then parses the sample description for the numeric data and passes this data along with the corresponding radioactivity to the plotting subroutine. Options exist for adding titles, labels, colours and scaling the $x$ and $y$ axes.

### Discussion

LSCAS has been used successfully in our laboratory to perform data capture and data-base management. Most of the application programs being developed will not reside on the Perkin-Elmer because of the excellent data-base and statistical package currently available on the VAX. A wide selection of statistical routines allow the user to perform many mathematical and statistical data calculations with little or no programmer intervention. Database software (for example SMARTSTAR [2]) and statistical software (for example SAS [3]) available on larger computers such as the VAX 11/750, are not available on the Perkin-Elmer 3230. Fortunately the strengths of both the VAX 11/750 and the Perkin-Elmer 3230 can be utilized when the two computers are allowed to communicate with each other.

Currently, data transfer between the Perkin-Elmer 3230 and the VAX 11/750 is provided by means of a VAX 3780 Protocol emulator. This emulator can be invoked interactively or by a command procedure. Major features of the emulator include 3780 binary synchronous communications, 9600 bits/second line speed and binary or ASCII data types. In LSCAS, the Fortran program retrieves the data and formats it according to the particular application program requirements. LSCAS then exits to the operating system and sends the data to a batch queue. Thus, the data transfer protocol operating in the background provides the user with the resources of the LSCAS while the data is being transferred to the VAX 11/750.

### References

1. Interfacing your Beckman nuclear counter to a computer. *Nuclear Counting Exchange*, **1**, 19.
2. SMARTSTAR is a registered trademark of Signal Technology, Inc., Goleta, California, USA.
3. SAS is a registered trademark of SAS Institute, Inc., Cary, North Carolina, USA.

